# Anti-proliferative, pro-apoptotic and anti-invasive effect of the copper coordination compound Cas III-La through the induction of reactive oxygen species and regulation of Wnt/β-catenin pathway in glioma

**DOI:** 10.7150/jca.59769

**Published:** 2021-07-25

**Authors:** Rosa Angélica Castillo-Rodríguez, Guadalupe Palencia, Isabel Anaya-Rubio, Juan Carlos Gallardo- Pérez, Dolores Jiménez-Farfán, Ángel Escamilla-Ramírez, Sergio Zavala-Vega, Arturo Cruz-Salgado, Claudia Cervantes-Rebolledo, Isabel Gracia-Mora, Lena Ruiz-Azuara, Cristina Trejo-Solis

**Affiliations:** 1 CONACYT-Instituto Nacional de Pediatría, Ciudad de México 04530, México; 2 Laboratorio Experimental de Enfermedades Neurodegenerativas, Instituto Nacional de Neurología y Neurocirugía, Ciudad de México 14269, Tlalpan, México; 3 Departamento de Bioquímica, Instituto Nacional de Cardiología, Ciudad de México 14080, Tlalpan, México; 4 Laboratorio de Inmunología, División de Estudios de Posgrado e Investigación, Facultad de Odontología, Universidad Nacional Autónoma de México, Ciudad de México 04510, México; 5 Hospital Regional de Alta Especialidad de Oaxaca, Secretaria de Salud, C.P. 71256 Oaxaca, México; 6 Departamento de Patología, Instituto Nacional de Neurología y Neurocirugía, Ciudad de México 14269, Tlalpan, México; 7 Universidad de Ixtlahuaca-UICUI, México, Estado de México; 8 Departamento de Química Inorgánica y Nuclear, Facultad de Química, Universidad Nacional Autónoma de México (UNAM), Ciudad Universitaria, Investigación Científica 70, Ciudad de México 04510, México; 9 Facultad de Química, Departamento de Química Inorgánica y Nuclear, Universidad Nacional Autónoma de México, Ciudad de México 04510, México

**Keywords:** glioma cells, Cas III-La, reactive oxygen species, GSK3β, β-catenin, apoptosis

## Abstract

Gliomas are the most aggressive neoplasms that affect the central nervous system, being glioblastoma multiforme (GBM) the most malignant. The resistance of GBM to therapies is attributed to its high rate of cell proliferation, angiogenesis, invasion, and resistance to apoptosis; thus, finding alternative therapeutic approaches is vital. In this work, the anti-proliferative, pro-apoptotic, and anti-invasive effect of the copper coordination compound Casiopeina III-La (Cas III-La) on human U373 MG cells was determined *in vitro* and *in vivo*. Our results indicate that Cas III-La exerts an anti-proliferative effect, promoting apoptotic cell death and inactivating the invasive process by generating reactive oxygen species (ROS), inactivating GSK3β, activating JNK and ERK, and promoting the nuclear accumulation of β-catenin. The inhibition of ROS generation by N-acetyl-l-cysteine not only recovered cell migration and viability, but also reduced β-catenin accumulation and JNK and ERK activation. Additionally, Cas III-La significantly reduced tumor volume, cell proliferation and mitotic indices, and increased the apoptotic index in mice xenotransplanted with U373 glioma cells. Thus, Cas III-La is a promising agent to treat GBM.

## Introduction

Gliomas are the most common primary tumors affecting the central nervous system (CNS) 1. As the name implies, they are derived from glial cells. About 2% of all malignant tumors are CNS primary neoplasms, and 80% of these tumors are of glial origin. The incidence rate of gliomas is 5.73/100 000 2. The World Health Organization (WHO 2016) categorizes gliomas into four grades according to histopathological traits and genetic markers 3, being anaplastic astrocytoma (grade III) and glioblastoma multiforme (GBM; grade IV) the types with the highest degree of malignancy 3, 4. The frequently observed resistance of GBM to several therapies has been attributed to its high rate of cell proliferation, angiogenesis, and invasion, as well as their intense resistance to apoptosis 5. Additionally, this neoplasm exhibits a high degree of invasiveness to the brain parenchyma. This migration, usually within 1-2 cm from the original tumor mass, often prevents a total tumor resection after surgery and results in a high rate of tumor recurrence 6. Several epidemiological studies have reported survival times less than two years after diagnosis despite surgery, radiotherapy, chemotherapy, or a combined treatment 7. Thus, studying the pathophysiology of GBM and identifying new alternative treatments is of utmost importance.

Casiopeinas are coordination compounds with copper (II) as the metallic center, with a coordination sphere conformed by substituted diamines like 1,10-phenanthroline (X-phen) or 2,2'-bipyridine (X-bpy) as a primary ligand and an acetylacetonate (O-O) or amino acid anion (N-O) as a secondary ligand. Here, the “primary” and “secondary” tags indicate a ligand's capability to modulate the physicochemical properties of the coordination compound. They have shown antitumor activity in various cancer models, both *in vitro* and *in vivo* 8-11. It has been suggested that Casiopeinas containing phenanthroline or bipyridine as primary ligands and acetylacetonate as a secondary ligand, such as Cas III-La ([Cu(5,6-dimethyl- 1,10-phenantroline) (acetylacetonate)]·H_2_O·NO_3_), is more potent as antineoplastic drugs due to an increased permeability than those containing glycinate instead of acetylacetonate, such as Cas IIgly ([Cu(4,7-dimethyl- 1,10-phenanthroline) (glycinate)]·H_2_O·NO_3_), and those whose imine ligand is bipyridine, such as Cas III-ia ([Cu(4,4'-dimethyl-2,2-bipyridine)(acetylacetonate)]·H_2_O·NO_2_) 12. The mechanisms through which Casiopeinas exert their anti-tumor effect have been studied, showing that they induce an redox imbalance, and it has been proposed that reactive oxygen species (ROS) could mediate it 13-16. ROS have been reported as involved in the induction of apoptosis through the oxidation of lipids, proteins, and nucleic acids. They also play a role in cell death by regulating signaling molecules, including serine/threonine kinases (PI3K, Akt, JNK, ERK) and transcription factors (AP-1, FOXO, NF-κB) 17, 18. The evidence suggests that Cas II-gly and Cas III-ia induce an apoptotic effect in rat glioma cells through the generation of ROS and an activation of the c-jun NH_2_-terminal kinase (JNK) 17, 18. Bravo et al. provided a chemical, physicochemical, and biological characterization of several Casiopeinas, including Cas III-La, and demonstrated that Cas III-La showed a more pronounced anti-proliferative activity than Cas III-ia and Cas II-gly on MCF-7 breast cancer cells, HCT-15 colorectal cancer cells, HeLa cervix cancer cells, and the SiHa squamous cervix cancer cell line. These authors also suggested that Cas III-La induces the oxidation of glutathione in the presence of hydrogen peroxide, or that molecular oxygen generates hydroxyl radical and superoxide overproduction, inducing oxidative toxicity *in vitro* and *in vivo* 19. Additionally, it has been reported that Casiopeinas with extended aromatic systems (phenanthroline instead of bipyridine) such as Cas III-La have a more suitable redox potential to participate in the aforementioned glutathione oxidation 20. In addition, results of a molecular dynamics study suggest that the topology and effective volume of Cas III-La allows it to cross cellular membranes by a diffusion process more easily than its analog Cas III-Ea ([Cu(4,7-dimethyl-1,10-phenanthroline)(acetylacetonate)]NO_3_). Our results allow us to explain not only the differences seen in the antiproliferative activity of the drug on phagocytic (macrophages) and non-phagocytic (lymphocytes) cells, but also the completely different pharmacodynamic parameters observed for Cas III-La and CasIII-Ea 21.

Furthermore, it has been reported that H_2_O_2_ regulates the activity of Wnt/β-catenin 22, 23. The canonic Wnt/β-catenin pathway participates in several cellular processes, including embryonic development and cell growth, invasion, and differentiation, but also in cell cycle arrest and apoptosis 24, 25. In the absence of Wnt activators, free cytosolic β-catenin is degraded by the CKI/GSK3β/Axin/APC destruction complex. β-Catenin is sequestered by Axin, favoring its phosphorylation, first at Ser^45^ by CKI and then at Ser^33,37^ by GSK3β. Phosphorylated β-catenin is ubiquitinated and recognized by proteasomes 26. When Wnt is stimulated, it binds the Frizzled and LRP5/6 receptors. Frizzled recruits and phosphorylates Dishevelled (Dvl), which interacts with Axin (bound to LRP5/6) and Frat1/GBP to inhibit GSK3β, resulting in the accumulation of β-catenin and its translocation into the nucleus, where it binds and acts as a co-activator for transcription factors of the TCF/LEF1 family, inducing genic expression 27. Several studies have reported the role of Wnt/β-catenin in promoting cell death by apoptosis, despite the highly oncogenic effect of its activation 24, 25. Ming et al. reported that the overexpression of Wnt/β-catenin signaling in hematopoietic progenitor cells triggered the mitochondrial apoptotic pathway by decreasing the expression of the anti-apoptotic protein Bcl-2 and activating the executor caspase-3 28. Additionally, the activation of Wnt/β-catenin via wnt3A has been reported to increase the sensitivity of the recombinant TNF receptor death-inducing ligand (rTRAIL) to apoptosis on melanoma cells through upregulation of the pro-apoptotic proteins Bim and Puma, and the downregulation of the anti-apoptotic protein Mcl-1 29. In addition, Zhang et al. demonstrated that the overactivation of β-catenin downregulates the caspase-8 inhibitor c-FLIP, promoting the activation of the apoptotic pathway via death receptors in human colon polyps treated with rTRAIL 30. It has been suggested that, depending on its levels, β-catenin can induce cell death or favor the oncogenic process 22, 23. Gao et al. demonstrated that the inhibition or stimulation of the Wnt/β-catenin pathway can reduce the proliferation and invasion in U87 cells 31.

To determine whether Cas III-La induces antineoplastic effects via ROS generation and by modulating signaling molecules, we analyzed for the first time the antiproliferative, pro-apoptotic, and anti-invasive effects of the copper coordination compound Cas III-La on U373 MG glioma cells, in vitro and in vivo. Our work demonstrates that this compound induces apoptotic cell death and shows anti-migratory and anti-invasive capacities by generating reactive species, which lead to GSK3β inactivation and the activation of JNK and ERK, as well as the nuclear accumulation of β-catenin. Additionally, this is the first report to demonstrate that β-catenin accumulation has a pro-apoptotic effect on U373 MG glioma cells.

## Materials and Methods

### Synthesis of Casiopeina III-La

Casiopeina III-La was synthetized as previously described in patents 19, dissolved in double-distilled water, and filter-sterilized.

### Glioma cell culture

U373 MG human glioma cell cultures (American Type Culture Collection, Rockville, Maryland, USA), were kept at 37 °C and 5% CO_2_ under sterile conditions in Dulbecco's modified Eagle's medium (DMEM, Sigma Chemical, St. Louis, MO, USA), supplemented with 10% fetal bovine serum (FBS, Gibco, Thermo Fisher Scientific, Waltham, MA, USA) plus 1% antibiotics (Gibco), and treated for 24 h with 1.5 μg/ml (3.32 μM) or 2.5 μg/ml (5.54 μM) of Cas III-La, either with or without N-acetyl-L-cysteine (NAC) 5 mM and Akt Inhibitor IV 10 μM.

### Cell viability

The effect of Cas III-La on the survival of U373 human glioma cells was determined by the MTT assay (3-[4,5-dimethylthiazol-2-yl]-2,5-diphenyltetrazolium bromide), using the Cell Proliferation Kit I (Roche Diagnostics, Mannheim, Germany), to measure mitochondrial activity. The cells were seeded in a 96-well plate at a density of 10^5^ cells/well in DMEM. Twenty-four hours later, the cells were treated with Cas III-La at the concentrations described above; untreated cells were used as controls. After incubation for 24 h, a cell viability assay was performed as described somewhere else 32.

### Apoptosis assessment

To evaluate cell death on U373 MG human glioma cells after exposure to Cas III-La, the In Situ Cell Death Detection Kit (Roche Diagnostics) was used. Briefly, 1.5 × 10^5^ cells were seeded in 8-well culture plates. After Cas III-La treatment, the cells were washed twice with phosphate buffer solution (PBS) and fixed with 3.7% paraformaldehyde (PFA), washed with PBS again, permeabilized with sodium citrate 0.1 M (pH 6), and incubated in the TUNEL reaction mixture for 1 h at 37 °C. The plates were washed three times with PBS, and the slides were mounted in Vectashield mounting medium with DAPI. The cells were observed under an Olympus inverted microscope (objective 20x), and microphotographs were taken with the software Olympus FluoView v.1.7. Cell death was also determined by the appearance of the sub-G_0_ peak in cell cycle analysis. Briefly, 10^6^ control and treated cells were centrifuged and fixed overnight in 70% ethanol at 4 °C; then, the cells were washed three times with PBS, incubated for 1 h in the presence of 1 mg/ml of RNase A and 20 µg/ml of propidium iodide at room temperature, and analyzed for different cell cycle phases with a FACScan flow cytometer (Becton Dickinson, San Jose, CA, USA).

### Western blot

The samples (50 µg) corresponding to total cell lysates were run on 10-12% SDS-PAGE gels and transferred to nitrocellulose membranes. The membranes were blocked and incubated with the respective primary antibody to a 1:500 final dilution (PCNA, Bcl-2, pBcl-2 (Ser^87^), Bcl-xL, Bid, Bax, caspase-3, caspase-8, caspase-9, pGSK3β (Ser^9^), GSK3β, and pβ-catenin (Ser^45^), pβ-catenin (Ser^33/37^), β-catenin, pJNK (Thr^183^ and Tyr^185^), JNK, pERK (Tyr^204^), ERK, AKT, pAKT (Ser473), MMP2, MMP9, SNAIL, E-cadherin, vimentin fibronectin, cytokeratin, β-actin, and β-tubulin (Santa Cruz Biotechnology, Santa Cruz, CA, USA, and Abcam) for 24 h at 4 °C. Immunoreactivity was visualized by probing with a horseradish peroxidase-conjugated secondary antibody (Santa Cruz Biotechnology) and detected using the ECL kit (Santa Cruz Biotechnology).

### Determination of Mitochondrial Membrane Potential (mΔψ)

The changes in mitochondrial membrane potential (∆Ψm) were observed by using JC-1. U373 cells were seeded into an 8-well plate and incubated with Cas III-La. After 24 h, the cells were washed twice with Hanks' solution and incubated with 10 µg/ml of JC-1 dye at 37°C for 30 min. Then, the cells were washed with Hanks' solution twice and observed under a Fluoview FV1000 inverted laser scanning confocal microscope (Olympus, Tokyo, Japan).

### Immunofluorescence assay

Immunocytochemistry assays were performed on cells seeded in 8-well plates (Daigger Scientific, Vernon Hills, IL, USA) as reported previously. The cells were fixed with 3.7% PFA in PBS at 4 °C, washed, permeabilized with DAKO target retrieval solution (Dako Cytomation, Carpinteria, CA, USA), and blocked with 2% albumin at room temperature. Then, the cells were incubated with monoclonal antibody [MMP2, MMP9, pGSK3β (Ser^9^), GSK3β, and pβ-catenin (Ser^45^), pβ-catenin (Ser^33/37^), β-catenin, pJNK (Thr^183^ and Tyr185), JNK, pERK (Tyr^204^), ERK] diluted at 1:100 (Santa Cruz Biotechnology) for 1 h at room temperature and developed either with FITC or rhodamine-conjugated secondary antibody (Jackson ImmunoResearch Laboratories, West Grove, PA, USA) at a 1:200 dilution. The slides were mounted in DAPI solution (Vector Laboratories, Inc. Burlingame, CA, USA) and observed under a Fluoview FV1000 inverted laser scanning confocal microscope (Olympus).

### Determination of reactive oxygen species

The DCFH-DA probe was used for ROS determination as described somewhere else 33. Treated and control cells were lysed and diluted 1:10 with Tris 40 mM (pH 7.4) plus DCFH-DA (2′,7′-dichlorofluorescein diacetate; Molecular Probes, Eugene, OR, USA) 5 µM in methanol for 15 min at 37 °C. Fluorescence intensity was measured before and after 60 minutes of incubation in an LS50-B luminescence spectrometer (Perkin-Elmer, Boston, MA, USA). The reaction with superoxide anion, hydrogen peroxide, and hydroxyl radical to produce an oxidized, fluorescent DCFH derivative named DCF was monitored at an excitation wavelength of 525 nm (slit width = 5 nm). The bucket holder was thermostatically kept at 37 °C. Autofluorescence of the cellular lysate was less than 6% at any time. The fluorescent signals of both methanol (vehicle) and the substrates were recorded at the baseline, before DCF formation. DCF was quantified from a standard curve (Sigma Aldrich, St. Louis, MO, USA) in methanol.

### Localization of superoxide in mitochondria

To detect the presence of mitochondrial superoxide by confocal microscope, the cells were seeded in 8-well plates for 24 h. After Cas III-La treatment, treated and control cells were washed twice with Hanks' solution and incubated with MitoSOXTM red indicator 5 µM (Molecular Probes, Eugene, OR, USA) for 15 minutes. When oxidized by superoxide, the indicator emits fluorescence upon excitation. The assay was performed following the manufacturer's instructions.

### Localization of reactive oxygen species in mitochondria

Treated and control cells were washed with PBS and incubated with DCFH-DA for 20 min at 37 °C. Then, the cells were washed with PBS and incubated with 500 nm of MitoTracker probe (Molecular Probes) for 30 min, following the manufacturer's instructions. Fluorescence intensity was observed directly under a Fluoview FV1000 inverted laser scanning confocal microscope (Olympus).

### Wound-healing assay

Cell migration was assessed by the wound-healing migration assay, observed under a light microscope. About 10^6^ U373 MG cells were seeded in 6-well plates. A wound was made on the monolayer using a sterile micropipette yellow tip and the medium was removed; fresh medium was added plus Cas III-La at concentrations of 0.75 and 1.5 μg/ml with or without 5mM NAC. Plate microphotographs were taken at time 0 and 24 h after treatment.

### Invasion assay

The invasion capacity of U373 MG human glioma cells after treatment with Cas III-La was evaluated with the fluorometric QCM 24-Well Cell Invasion Assay (Chemicon International, Burlington, MA, USA). About 1.25 × 10^5^ cells were seeded in the 24-well plate inserts of the Cell Invasion kit in FBS-free DMEM. Treated groups were added with 0.75 or 1.5 μg/ml of Cas III-La. The lower chamber was added with 500 µl of DMEM supplemented with 10% FBS as a chemoattractant for invasive U373 MG glioma cells. The plate was incubated for 24 h at 37 °C under 5% CO2. After incubation, the inserts were placed in a new plate and each one was added with 225 μl of Cell Detachment Solution and incubated for 30 min at 37 °C. Then, 75 μl of Lysis Buffer/CyQUANT GR dye were added to each well and incubated for 15 min at room temperature. Thereafter, 200 μl of each sample were transferred to a 96-well fluorescence plate and read at 480/520 nm.

### Zymograms

MMP2 and MMP9 activity was observed by a gelatin zymography assay. About 10^6^ cells were seeded in 6-well plates and treated with FBS-free DMEM plus Cas III-La at the concentrations mentioned above. After a 24 h treatment, the supernatant was collected and analyzed. Gelatin zymography was performed on SDS-polyacrylamide gels, copolymerized with 1% gelatin as described somewhere else 34. After electrophoresis, the gels were washed with Triton X-100 (2.5%) for 90 min and incubated for 48 h in buffer (5 mM CaCl_2_, 50 mM Tris-HCl (pH 8), Triton X-100 (2.5%)). The gels were stained with 0.5% Coomassie brilliant blue R-250. MMP2 and -9 activity was detected as translucent bands over the blue background of blue stained gel.

### Tumor model and treatment

Fourteen male, 6 weeks-old nude mice (nu/nu) (Harlan Inc, Indianapolis, IN, USA) were kept under specific pathogen-free conditions with free access to autoclaved food and water. All mice were injected subcutaneously with 10^6^ U373 cells in the low left flank. When the tumor reached a 0.3-cm diameter, the mice were randomly distributed into a control group (*n* = 7), injected subcutaneously with 0.5 ml of water every 24 h for 21 days, and a treated group (*n* = 7), intraperitoneally injected with 0.8 mg/kg of Cas III-La every 24 h for 21 days. All mice were treated in accordance to NOM-062-ZOO-2001 (guidelines for the care and use of laboratory animals).

### Antineoplastic evaluation in vivo

Twenty-one days after treatment, the mice from both groups were humanely killed. The tumors were measured every day in length and width using Vernier calipers. Tumor volume was calculated as [length (cm) × width^2^ (cm) × *p*]/6. Then, the tumors were dissected and cut by the middle into eight parts; the sections were stained by the hematoxylin and eosin method for morphological study. The mitotic index was determined as the mean percentage of mitoses in 10 different fields. For cell proliferation studies, the sections were stained by immunohistochemistry with monoclonal antibodies against PCNA (DAKO). Cell proliferation index was determined by the mean number of positive cells in 10 different microscopic fields. All histologic evaluations (x40) were made blindly to prevent bias. Apoptosis determination in 3-µm U373 glioma sections was also analyzed using the InSitu Cell Death Detection Kit, AP (Roche Diagnostics) as described above. The fraction of apoptotic cells was expressed as an apoptotic rate, the number of apoptotic cells in 1000 glioma U373 nucleated cells.

### Statistical analysis

All experiments were performed in triplicate. Data from experiments were analyzed by two-way ANOVA, followed by a Tukey multiple comparison test. *P* < 0.05 was regarded as significant.

## Results

### Cas III-La decreased cell viability and proliferation

**Fig 1:**
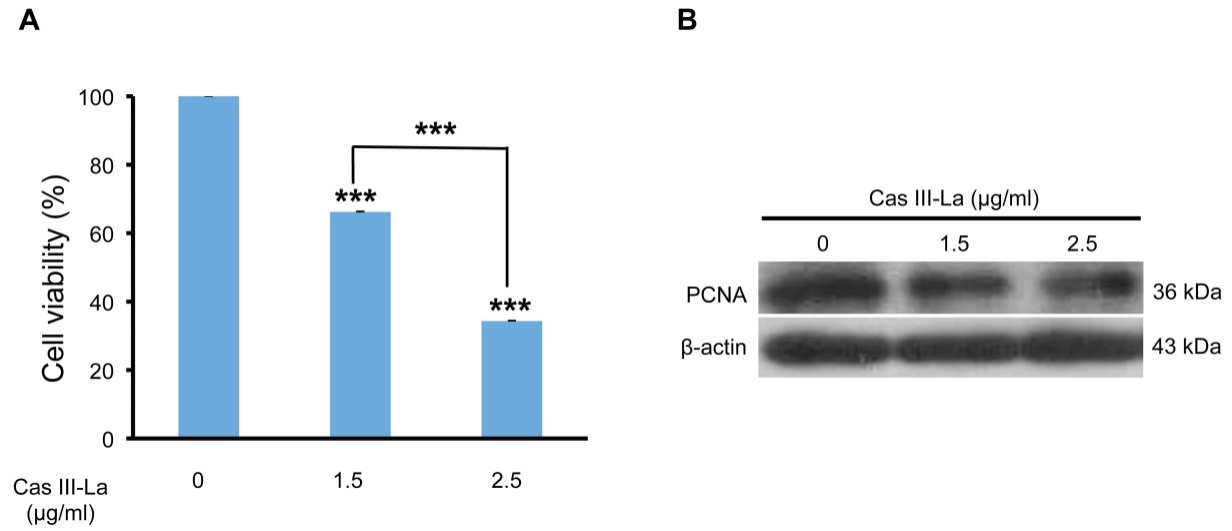
** Cas III-La inhibits the cell proliferation in U373 glioma cells**. (**A**) Dose-dependent effect of Cas III-La on cell viability in U373 cells. Cell viability was measured by the MTT assay; data represent the mean ± SD (*P ≤ 0.05, **P ≤ 0.001, and ***P ≤ 0.0001) from three independent experiments. (**B**) Dose-dependent effect on the expression of PCNA in cell lysates of control and Cas III-La treated glioma U373 cells (right panel). The figure is representative of at least three different experiments for each experimental condition.

To analyze the viability of the U373 MG cell line in the presence of Cas III-La, an MTT colorimetric assay was performed. Cell cultures were treated with Cas III-La at a concentration of 0.5, 0.75, 1.5, and 2.5 µg/ml and incubated for 24 h; control cells were added only with DMEM plus FBS. A dose-dependent decrease in cell viability of 34% and 66% with respect to controls was observed in the group treated with Casiopeina at a dose of 1.5 µg/ml and 2.5 µg/ml , respectively; these differences were statistically significant (Fig. 1A). However, Cas III-La failed to show a significant effect on cell viability with respect to controls at a dose of 0.5 or 0.75 µg/ml (data not shown). A mean inhibitory concentration (IC_50_) of 1.89 µg/ml was determined for Cas III-La on U373 GM cells.

On the other hand, the expression of the proliferating cell nuclear antigen (PCNA) was analyzed by Western blot as a marker of DNA replication, and therefore proliferation. We found that PCNA levels were significantly decreased with respect to the control group when the cells were treated with 1.5 or 2.5 µg/ml of Cas III-La (Fig. 1B). These results demonstrate the antineoplastic effect of Cas III-La on U373 MG cells by inhibiting cell proliferation.

### Cas III-La induced cell death

To determine whether the observed decrease in cell viability by Cas III-La was due to cell death induction, U373 cells were treated with 1.5 or 2.5 µg/ml of Cas III-La for 24 h, stained with propidium iodide, and analyzed by flow cytometry. As shown in Table 1, Cas III-La increased cell death rate in a dose-dependent manner. After treatment with 1.5 or 2.5 μg/ml of Cas III-La, 43.29% and 73.36% of U373-MG cells were in sub-G_0_ phase, respectively, whereas only 2.48% of cells in control samples were in sub-G_0_. Flow cytometry analysis also indicated that Casiopeina significantly diminished the fraction of cells in the G_0_/G_1_ and S/M phases at both concentrations. These results suggest that Cas III-La leads to cell death irrespectively of the cell cycle phase.

### Cas III-La induced apoptosis

**Fig 2:**
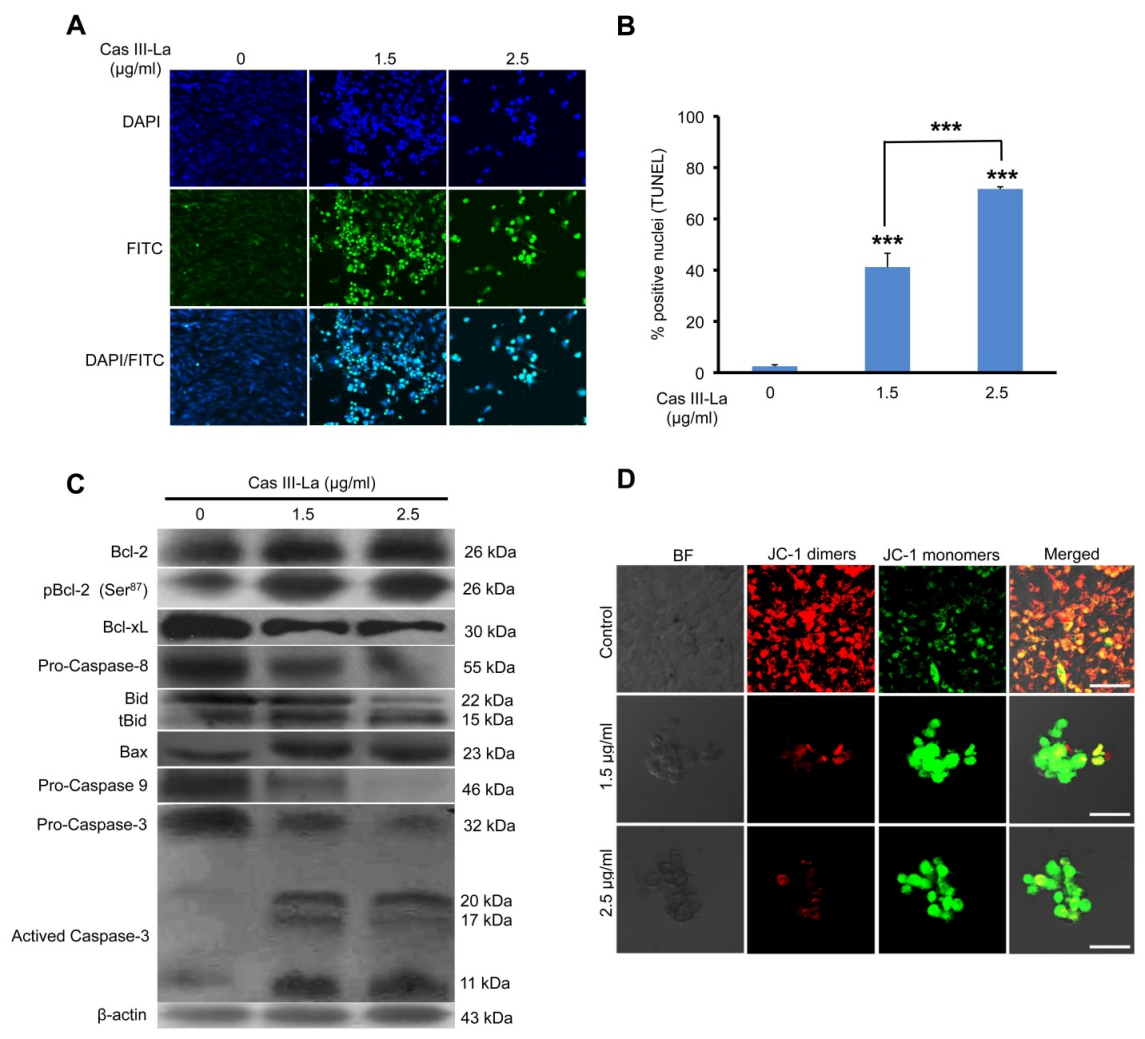
** Cas III-La induces apoptosis and depolarization of the mitochondrial membrane.** (**A**) Apoptosis was determined by the TUNEL assay and visualized by confocal microscope (Green-fluorescein and Blue-DAPI) in untreated U373 cells (controls) and cells treated with Cas III-La (1.5 or 2.5 μg/ml) for 24 h. Original magnification x20. (**B**) A quantitative estimation of TUNEL-positive nuclei at different doses was performed by counting five fields at x10 for each determination. The results are expressed as the mean ± SD of three independent determinations (*P ≤ 0.05 and ***P ≤ 0.0001). (**C**) Expression of the proteins Bcl-2, pBcl-2, Bcl-xL, Bid, Bax, procapases-8, -9 and 3 in control and treated glioma cells determined by Western blot. (**D**) As mitochondria membrane depolarization levels increase, fluorescence by JC-1 monomers (green) in cells treated with 1.5 or 2.5 μg/ml of Casiopeina III-La. Control cells show a predominant JC-1 dimer fluorescence, corresponding to alive cells (bar = 50 μm). All figures are representative of at least three different experiments for each experimental condition.

To investigate whether cell death due to Cas III-La treatment was caused by apoptosis, the proportion of apoptotic cells was determined by a TUNEL assay. As seen in Fig. 2A, FITC-labeled fragmented DNA in apoptotic cells is overlapping with the nuclear marker DAPI. After treatment with 1.5 and 2.5 μg/ml of Cas III-La, 41% and 72% of cells were apoptotic, respectively, while only 2% of apoptotic cells were observed in controls.

Then, the possible participation of members of the Bcl-2 family like Bcl-2, Bcl-xL, Bid, and Bax, as well as caspase-8 (the initiating caspase in the death receptor pathway), caspase-9 (the initiating caspase in the mitochondrial pathway), and caspase-3 in the antineoplastic effect of Cas III-La on U373 MG cells was studied by Western blot. As shown in Fig. 2B, treatment with Cas III-La at a dose of 1.5 or 2.5 µg/ml decreased the levels of the anti-apoptotic protein Bcl-xL, increased the levels of the anti-apoptotic protein Bcl-2, and also decreased the levels of the phosphorylated, inactive form of Bcl-2 (pBcl-2). An increase in the expression of tBid (truncated Bid), Bax, and activated caspase-3 was also detected in treated groups with respect to controls. Additionally, decreased levels of procaspase-8 and -9 were observed in U373 cells treated with Cas III-La with respect to controls, suggesting the possible activation of these caspases. No active fragments of both caspases were detected, since only antibodies recognizing the respective procaspase were used. Furthermore, we observed a greater increase in the rate of cleavage of the pro-apoptotic protein Bid to tBid induced by Cas III-La at both doses, in contrast with controls. It has been demonstrated that, in response to an apoptotic signal, Bid is cleaved by caspase 8 to yield the C-terminal product tBid, which is then myristoylated and translocated to the mitochondria 35. These results suggest the participation of tBid, Bax, and caspases-8, -9 and -3 in the antineoplastic effect of Cas III-La on U373 MG glioma cells, as well as in the downregulation of Bcl-xL and the possible inhibition of Bcl-2 by its phosphorylation.

5,5',6,6'-tetrachloro-1,1',3,3'-tetraethyl-benzimidazolylcarbocyanine iodide (JC-1) is a cationic dye that probes mitochondrial depolarization, coloring cells either green (apoptotic) or red (living), an effect related to apoptosis induction 36. To assess possible changes in mitochondrial function after Cas III-La treatment, U373 cells were incubated with JC-1. As shown in Fig. 2C, a strong signal of JC-1 monomers (green) was observed in Casiopeina III-La-treated cells, indicating a change in mitochondrial membrane depolarization. On the other hand, JC-1 dimers (red, live cells) predominated in control cells. These results suggest that Cas III-La induces functional alterations, which can favor the mitochondrial apoptotic process through the release of cytochrome c (cyt c) and the activity of caspase-9.

### Cas III-La inhibited migration and invasion in vitro

**Fig 3:**
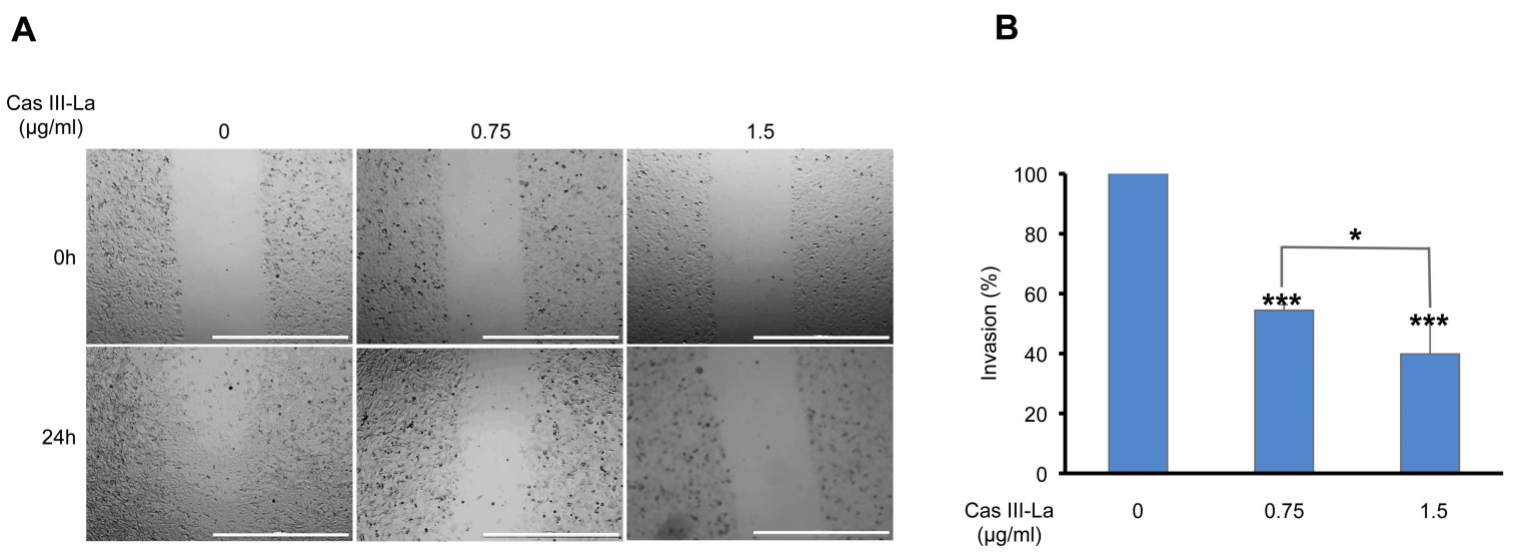
**Cas III-La inhibits cell migration and invasion in glioma cells.** (**A**) Cell migration was determined by wound healing in untreated U373 cells (controls) and cells treated with Cas III-La (0.75 or 1.5 μg/ml) for 24 h. The microphotographs are representative of at least three different experiments for each experimental condition. Bar = 1000 μm. (**B**) Cell invasion was determined by Transwell invasion assays for 24 h in untreated (control) and Cas III-La-treated U373 cells (0.75 and 1.5 μg/ml), respectively. Bars represent the mean ± SD of three replicates (P < 0.05, **P < 0.01, ***P < 0.001).

To determine the effects of Cas III-La on the migration of U373 cells, wound healing assays were performed. As shown in Fig. 3A, the migration capacity of U373 cells treated with 0.75 or 1.5 μg/ml of Cas III-La decreased significantly with respect to control cells. Then, the effect of Cas III-La on the invasive capacity of U373 MG cells was analyzed by Transwell assays. As shown in Fig. 3B, cell invasion decreased in a dose-dependent manner in the groups treated with Cas III-La with respect to control cells. The number of invasive cells diminished by 46% and 60% at a dose of 0.75 µg/ml and 1.5 µg/ml of Cas III-La, respectively, with respect to the control. Cell viability of invasive cells was also determined through an MTT assay. A 100% viability was observed in the control group and in cells treated with 0.75 µg/ml of Cas III-La, while viability decreased by 24% in the group treated with 1.5 µg/ml (data not shown). These results indicate that Cas III-La inhibits migration and invasion of U373 MG glioma cells.

### Cas III-La increased the expression of E-cadherin and reduced MMP activity

**Fig 4:**
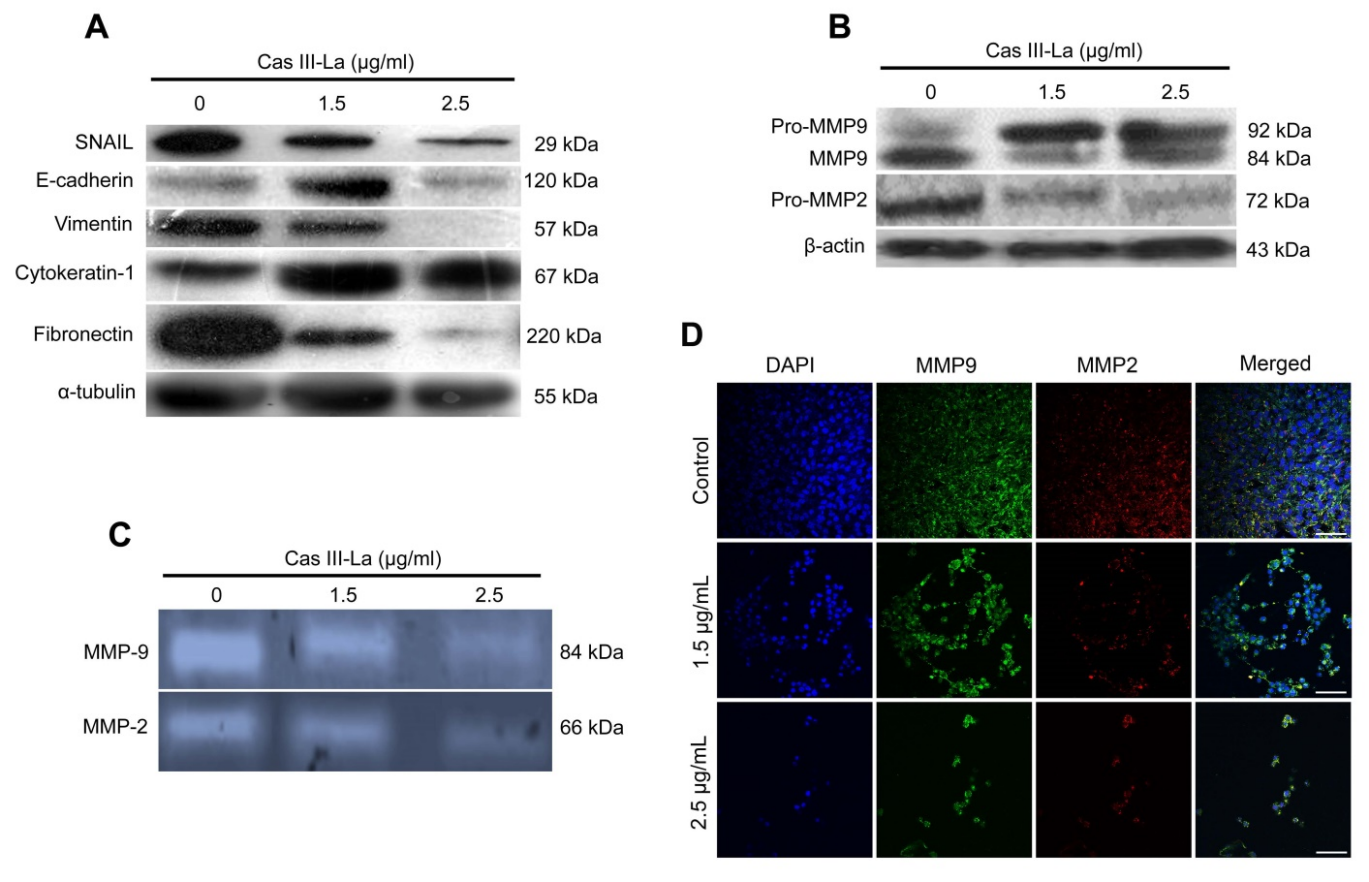
**Cas III-La inhibits the expression and activity of proteins involved in the invasive process of glioma.** (**A**) The expression of the proteins SNAIL, E-cadherin, vimentin, cytokeratin-1, fibronectin in untreated glioma cells (controls) and cells treated with Cas III-La (1.5 or 2.5 μg/ml) for 24 h was determined by Western blot. (**B**) The expression of the proteins MMP9 and MMP2 in untreated glioma (controls) and cells treated with Cas III-La (1.5 or 2.5 μg/ml) for 24 h was determined by Western blot. (**C**) MMP2 and MMP9 activity was determined by a gelatin zymography assay. (**D**) Immunocytochemistry of MMP9 (green-FITC and blue-DAPI) and MPP2 (red-rhodamine-blue-DAPI) in untreated glioma cells (controls) and cells treated with Cas III-La (1.5 or 2.5 μg/ml) for 24 h. All figures are representative of at least three different experiments for each experimental condition. Bar = 100 μm.

The GBM invasive phenotype is characterized by the loss of cell-cell and cell-extracellular matrix contact, and by a degradation of the basement membrane. In this study, the expression of proteins involved in these processes, like SNAIL, E-cadherin, vimentin, cytokeratin, fibronectin, matrix metalloproteinase-2 (MMP2), and MMP9 was determined by Western blot; the expression levels of these metalloproteases were also determined by immunofluorescence assays, and their activity was assessed by zymography. As shown in Fig. 4A*,* Cas III-La increased the levels of cytokeratin at both concentrations, as well as the levels of E-cadherin, but only at a dose of 1.5 µg/ml; conversely, both concentrations of Casiopeina led to significantly decreased levels of fibronectin, vimentin, and SNAIL, as determined by Western blot. MMP2 and MMP9 are well-documented endopeptidases, more specifically gelatinases, capable of degrading ECM components, and thus they are related with invasion and a worse prognosis in several cancer types 37, 38. As shown in Fig. 4B*,* Cas III-La decreased the levels of MMP9 and MMP2 with respect to controls; this pattern was corroborated by immunohistochemistry for pro-MMP9 and its active form (Fig. 4D). In addition, the activity of metalloproteases decreased, as observed in Fig. 4C. We concluded that Cas III-La induces the expression of adhesion markers but decreases the expression of proteins involved in the processes of migration and invasion.

### Cas III-La induced GSK3β inhibition, increased the expression and accumulation of β-catenin, and the activation of JNK and ERK

**Fig 5:**
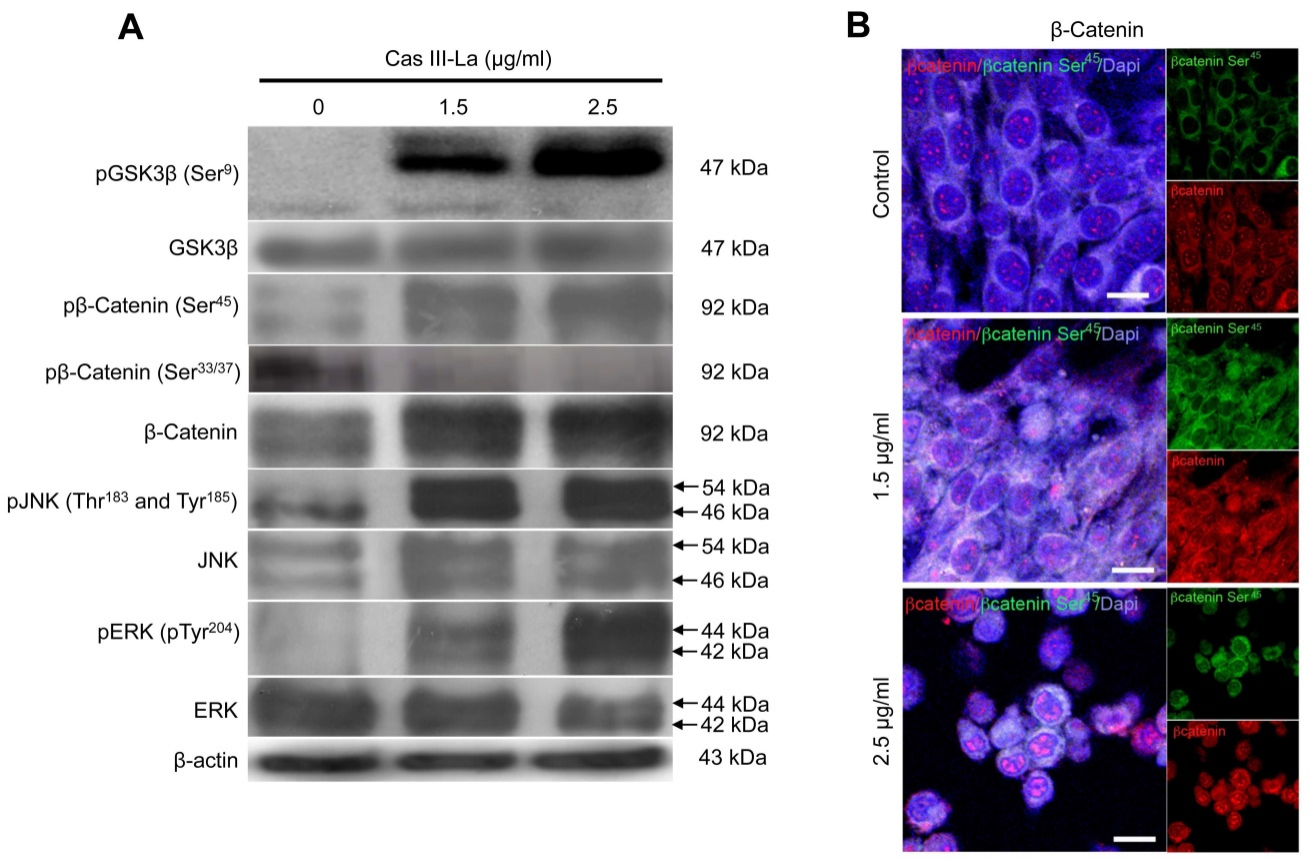
** Cas III-La induces inactivation of GSK-3β, nuclear accumulation of β-catenin, and phosphorylation of ERK and JNK.** (**A**) The expression of pGSK3β (Ser^9^), total GSK3β, pβ-catenin (Ser^45^ and Ser^33/37^), total β-catenin, pJNK (Thr^183^ and Tyr^185^), total JNK, pERK (Tyr^204^), and total ERK in untreated glioma cells (controls) and cells treated with Cas III-La (1.5 or 2.5 μg/ml) for 24 h was determined by Western blot. The figures are representative of at least three different experiments for each experimental condition; (**B**) The presence of total β-Catenin (rhodamine-red) increased in the nucleus in proportion to the dose of Cas III-La. A stronger signal of pβ-Catenin (Ser^45^) was detected in cytoplasm at a dose of 1.5 or 2.5 µg/ml of Cas III-La (green-FITC). Nuclei are counterstained with DAPI (blue). Bar = 12.5 μm.

β-Catenin, JNK, and ERK have been reported to play a role in the carcinogenic process in several neoplasms 18,19. To investigate the participation of β-catenin, JNK, and ERK in the antineoplastic effect induced by Cas III-La, the expression of pGSK3β (Ser^9^), GSK3β, pβ-catenin (Ser^45^ and Ser^33/37^), β-catenin, pJNK (Thr^183^ and Tyr^185^), JNK, pERK (Tyr^204^), and ERK was determined by Western blot (Fig. 5A) and immunofluorescence assays (Supplementary Fig. 1A). An increase in the levels of pGSK3β, β-catenin, and its phosphorylated form pβ-catenin (Ser^45^) were observed in cells treated with Cas III-La at a dose of 1.5 or 2.5 μg/ml with respect to controls. However, the levels of pβ-catenin (Ser^33/37^) were decreased in both doses of the drug, compared to the control (Fig. 5A and Supplementary Fig. 1A). On the other hand, an increase in phosphorylation levels of JNK and ERK were observed at both doses of Cas III-La. In contrast, Cas III-La had no effect on the levels of the proteins GSK3β, JNK, nor ERK (Fig. 5A and Supplementary Fig. 1A). Interestingly, β-catenin seemed to accumulate in the nucleus when the dose of Cas III-La was increased (Fig. 5B), but pβ-catenin (Ser^45^) did not. These results suggest that Cas III-La induces the inactivation of GSK3β, increasing the levels of pβ-Catenin (Ser^45^) and promoting the subsequent nuclear accumulation of β-catenin and the activation of JNK and ERK.

### Cas III-La did not inhibit the activity of Akt

**Fig 6:**
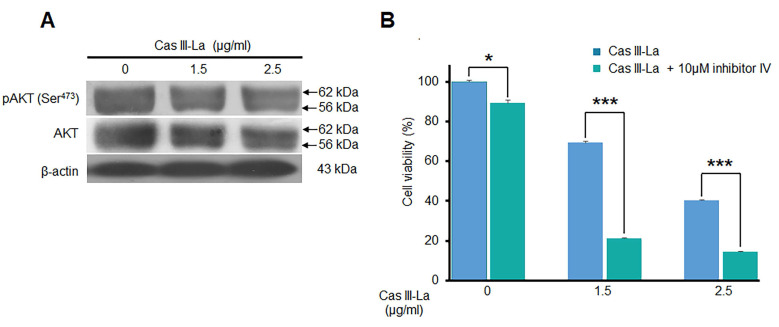
** Cas III-La showed no effect on Akt.** (**A**) The expression of pAkt (Ser^473^) and total Akt in untreated glioma cells (controls) and cells treated with Cas III-La (1.5 or 2.5 μg/ml) for 24 h was determined by Western blot. The figures are representative of at least three different experiments for each experimental condition; (**B**) The effect of Akt Inhibitor IV on cell viability in untreated glioma cells (controls) and cells treated with Cas III-La (1.5 or 2.5 μg/ml) for 24 h was determined by MTT assay; data represent the mean ± SD (*P ≤ 0.05, **P ≤ 0.001, and ***P ≤ 0.0001) from three independent experiments.

Akt is one of the main kinases that phosphorylate (inactivate) GSK3β at Ser^9^, leading to the nuclear translocation of β-catenin from the cytosol; thus, the effect of Cas III-La on Akt was determined by Western blot, detecting pAkt (Ser^473^) and Akt itself. As shown in Fig. 6A, Cas III-La failed to affect Akt activity, since no change was observed in Akt phosphorylation levels. Akt inhibitor IV had a synergic effect with Cas III-La at a dose of 1.5 or 2.5 μg/ml, as evinced by MTT viability assays (Fig. 6B).

### N-acetyl-L-cysteine inhibits Cas III-La anti-proliferative and-anti-migration effects

**Fig 7:**
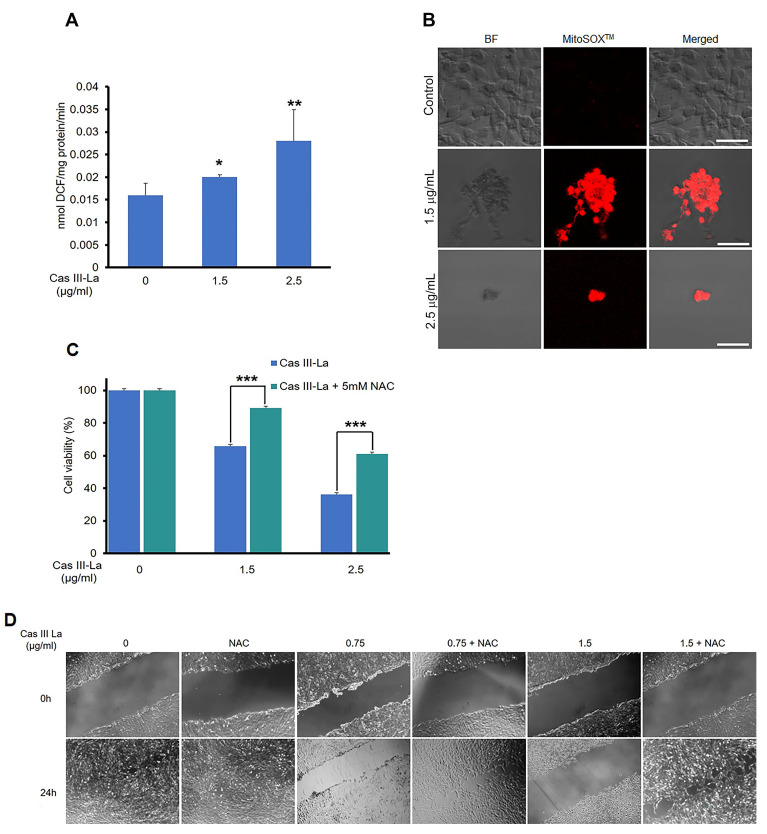
** NAC inhibits the anti-proliferative and-anti-migration effects of Cas III-La.** (**A**) ROS formation was determined in untreated U373 MG cells (controls) and cells treated with Cas III-La (1.5 or 2.5 μg/ml) for 24 h as described in the Materials and Methods section. Each bar represents the mean ± SD (*P ≤ 0.05, **P ≤ 0.001, and ***P ≤ 0.0001) from three independent experiments. (**B**) Dose-dependent effect of Cas III-La on mitochondrial superoxide formation in U373 cells, as detected by MitoSOX^TM^, a probe for this indicator. Bar = 50 μm. (**C**) Effect of NAC on cell viability in untreated glioma cells (controls) and treated with Cas III-La (1.5 or 2.5 μg/ml) for 24 h, with or without 5 mM NAC, was determined by an MTT assay; data represent the mean ± SD (*P ≤ 0.05, **P ≤ 0.001, and ***P ≤ 0.0001) from three independent experiments. (**D**) Cell migration was determined by wound healing in untreated U373 cells (controls) and cells treated with Cas III-La (0.75 or 1.5 μg/ml) with or without 5 mM NAC. The figures are representative of at least three different experiments for each experimental condition.

It has been reported that copper compounds induce the formation of ROS, which show antineoplastic effects on cancer cells 15; thus, we determined by fluorometric assay whether Cas III-La generated ROS, using DCFH-DA 33. Furthermore, we evaluated mitochondrial ROS production using the MitoSOX^TM^ red and carboxi-H_2_DCFDA/MitoTracker red fluorescent probes 33, 39. As shown in Fig. 7A, Cas III-La significantly induced the formation of ROS with respect to control cells; ROS were produced in mitochondria, as demonstrated by the increased intensity of red fluorescence (MitoSOX^TM^) in U373MG cells treated with Cas III-La (Fig. 7B). The presence of ROS in mitochondria was also evinced by the co-localization of green fluorescence (carboxi-H_2_DCFDA)/red fluorescence (MitoTracker red) in Cas III-La treated cells (Supplementary Fig. 2). Thus, a direct association can be established between Cas III-La treatment and the production of mitochondrial ROS. Then, the involvement of ROS in the antineoplastic effect of Cas III-La on U373 MG cells was assessed. The cells were preincubated either with or without a 5 mM solution of N-acetyl-L-cysteine (NAC), a ROS scavenger and antagonist, for 2 h, followed by Cas III-La treatment for 24 h. As shown in Fig. 7C, the addition of NAC blocked the anti-proliferative effect of Cas III-La at a dose of 1.5 or 2.5 µg/mL, significantly recovering cell viability. NAC also inhibited the anti-migratory effect of Cas III-La on U373 MG cells (Fig. 7D).

### NAC inhibited the accumulation of β-catenin and the activation of JNK and ERK

**Fig 8:**
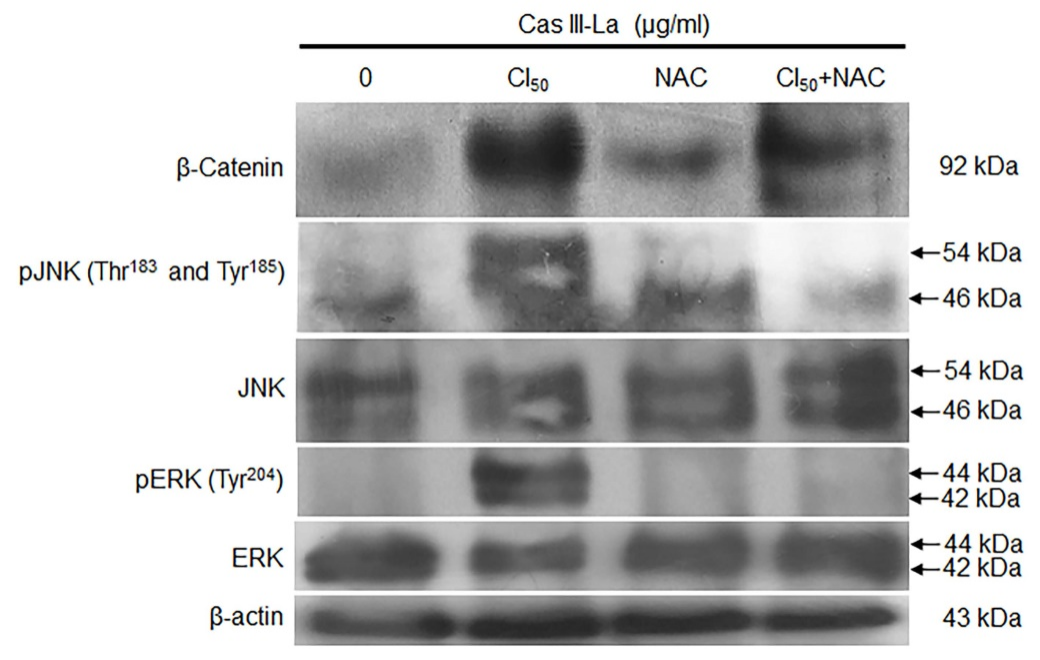
** NAC reduced the accumulation of β-catenin and the activation of JNK and ERK induced by Cas III-La.** The expression of β-catenin, pJNK, total JNK, pERK, and total ERK in untreated (control) cells and cells treated with 1.89 µg/ml of Cas III-La, 5 mM NAC, and Cas III-La + 5 mM NAC was determined by Western blot. The figures are representative of at least three different experiments for each experimental condition.

The effect of Cas III-La-generated ROS on β-catenin, JNK, and ERK was determined by Western blot. The induced increase in the levels of β-catenin, pJNK, and pERK by Cas III-La at the IC_50_ was decreased by cotreatment with NAC 5 mM plus 1.89 µg/ml of Cas III-La with respect to NAC-free cells (Fig. 8A). This pattern was corroborated by immunofluorescence assays (Supplementary Fig. 1B). These results suggest that ROS regulate the accumulation of β-catenin and the activation of JNK and ERK. It is also possible that ROS negatively regulate the GSK3β activity, since β-catenin phosphorylation (Ser^33/37^) is increased with cotreatment of Cas III-La plus NAC, as shown in the Supplementary Fig. 1B.

### Antitumoral effect of Cas III-La in vivo

**Fig 9:**
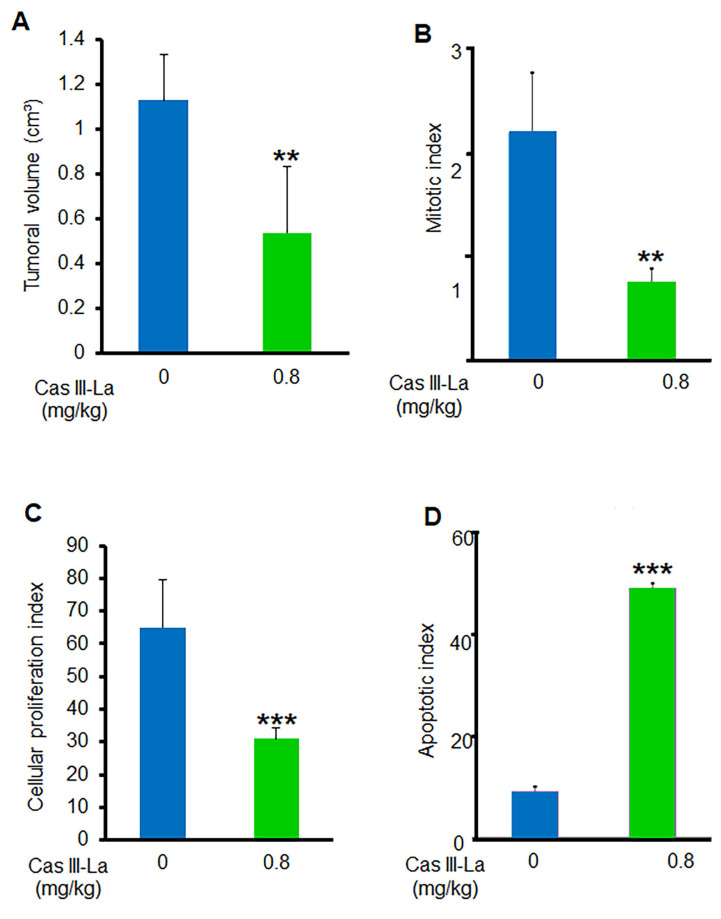
**Cas III-La showed an antitumoral effect in vivo.** (**A**) Tumor volume was calculated as [length (cm) × width^2^ (cm) × *p*]/6. (**B**) Mitotic index was determined by microscopic analysis. (**C**) Cellular proliferation index determined by immunohistochemistry for PCNA. (**D**) Apoptotic index determined by a TUNEL assay on nude mice xenotransplanted with the U373 MG cell line, treated with Cas III-La at a dose of 0.8 mg/kg per day for 21 days. Each bar represents the mean ± SD (*P ≤ 0.05, **P ≤ 0.001, and ***P ≤ 0.0001) from three independent experiments.

To determine whether Cas III-La exerts an antitumoral effect on nude mice xenotransplanted with the U373 MG cell line, tumor volume and the mitotic, proliferative, and apoptotic indices were assessed. A decrease by about 54% in the mean tumor volume was found in mice treated with 0.8 mg/kg/day of Cas III-La (Fig. 9*A*). With respect to the mitotic index, it was 2.12 ± 0.5 in viable sections of tumors from control mice, while a decrease of 65% was observed in treated animals with respect to controls (Fig. 9*B*). On the other hand, the cell proliferation index in animals treated with 0.8 mg/kg/day of Cas III-La was 31.5 ± 2.2, a decrease of 52% with respect to control mice (*P* ≤ 0.001) (Fig. 9*C*). Finally, the apoptotic index in treated mice was significantly higher than in controls (*P* ≤ 0.0001) (Fig. 9D). Significant differences were observed between treated animals and controls for all variables analyzed. No animals died during treatment.

## Discussion

The ability of tumor cells to proliferate and evade apoptosis is a key trait for their growth and invasive capacity. New chemotherapeutic agents with better anti-proliferative, pro-apoptotic, and anti-invasive effects are required to provide an effective treatment for GBM. In this work, we evaluate how Cas III-La, a novel member of the Casiopeina family, accomplished these goals on a model of U373 MG glioma cells. Cas III-La exerts an anti-proliferative effect on U373 MG cells, as corroborated by decreased PCNA levels; PCNA is a coactivator of DNA pol δ, an important molecule for DNA synthesis. In this sense, it is noteworthy that Cas III-La decreased significantly the fraction of cells in the G_0_/G_1_ and S/M phases.

Cas III-ia has been reported to increase cell death by apoptosis 16. This is consistent with our findings of increased levels of the pro-apoptotic proteins tBid and Bax, as well as the increased activity of caspase-8, -9, and -3. A decrease in the expression of Bcl-xL and the phosphorylation at Ser^87^ of the anti-apoptotic protein Bcl-2 was also observed. Post-translational modifications such as phosphorylation are known to regulate the activity of Bcl-2 40. Bcl-2 phosphorylation inhibits its binding to pro-apoptotic proteins like Bax, Bad, Bid, and Bim, thus facilitating the mitochondrial pathway to apoptosis 41. Furthermore, the phosphorylation of Ser^87^ in Bcl-2 has been reported as responsible for its proteasome-dependent degradation 42. JNK phosphorylates Bcl-2 at Thr^69^, Ser^70^, and Ser^87^ in response to microtubule-damaging agents, altering calcium concentrations in the endoplasmic reticulum, which induce apoptosis 41. In this study, Cas III-La was able to induce apoptosis in U373 MG cells either by activating death receptors or through the mitochondrial pathway, by inactivating anti-apoptotic proteins like Bcl-2 and Bcl-xL and activating pro-apoptotic proteins such as Bax, tBid, and the effector proteins caspase-8, -9, and -3. It has been demonstrated that the activation of caspase-8 through death receptors induces the activation of caspase-3, which in turn hydrolyzes components that are vital for cell proliferation, repair, and survival 43. Another target of caspase-8 is the pro-apoptotic protein Bid, which is hydrolyzed to a truncated, active form, which amplifies the apoptotic signal through the mitochondrial pathway by activating Bax and releasing cyt c into the cytosol, with the subsequent activation of caspase-9 and -3.

It has been widely reported that cell-cell union is mediated by proteins of the cadherin family 44. One of these proteins is E-cadherin, and its loss is considered one of the principal reasons of cell detachment from neighbor cells. It has also been demonstrated that the loss of proteins of the epithelial cell junction like E-cadherin and the simultaneous gain of mesenchymal markers like vimentin, fibronectin, and MMP2/MMP9 support the process of epithelial-to-mesenchymal transition (EMT) 44, which further promotes migration and invasion in epithelial tumors. In tumor cells, the transcription of E-cadherin is repressed by transcriptional factors with a zinc finger domain, such as SNAIL and TWIST, both of which direct the EMT 44, 45. In addition, E-cadherin is considered as a tumor suppressor due to its capacity of sequestering β-catenin. These data suggest that E-cadherin inhibits migration and invasion in tumoral cells. Decreased levels of E-cadherin have been related to a higher malignancy in gliomas 46. Additionally, a poorer prognosis in glioma has been related with an increase in SNAIL expression levels, which induce a down-regulation of E-cadherin and increase the expression of MMP2 and αvβ3-integrin 47. Irradiated glioma cells express high SNAIL levels, which promotes a transition from the glial to the mesenchymal state 48. On the other hand, it has been reported that when SNAIL expression is silenced in U87-MG and GBM05 glioblastoma-derived cell lines, the expression of vimentin is decreased, with the ensuing upregulation of E-cadherin and a decrease in proliferation, a retarded cell cycle, and impaired cell invasion/migration in vitro 47, 49. In malignant glioma cells, SNAIL has been reported to upregulate the expression of MMP2 and/or MMP9, which degrade type IV collagen in peripheral basement membranes to promote cell invasion 44, 50. The overexpression of MMP2 and MMP9 is related with proliferation, angiogenesis, and metastasis in glioblastoma 51, 52. We observed that Cas III-La on U373 cell line inhibited significantly wound healing and invasion, reducing the levels of SNAIL, vimentin, and fibronectin, as well as the expression and activity of MMP2 and MMP9. On the other hand, Cas III-La promotes the upregulation of E-cadherin only at a dose of 1.5 µg/ml, while it leads to a downregulation of this protein when administered at a dose of 2.5 µg/ml. Caspase-8 has been shown to activate c-Src, which phosphorylates E-cadherin, leading to its degradation 53. Zhou et al. reported that caspase-3 gene knockout in HCT116 and HT29 significantly increased E-cadherin expression 54. Additionally, E-cadherin has been reported to promote apoptosis via DR4/DR5 death receptors, increasing the activity of caspase-8 55. Thus, it is possible that Cas III-La promoted increased E-cadherin levels at a dose of 1.5 µg/ml, activating apoptosis, but down-regulated E-cadherin levels when administered at a dose of 2.5 µg/ml. Lu et al. demonstrated that epithelial-to-mesenchymal transition decreased apoptosis signaling via DR4/DR5 modulation, which inhibits the activity of caspase-8 and -3 55. These data suggest that Cas III-La inhibits EMT, which is necessary for wound healing, a key process for tumor migration/invasion. Therefore, the participation and regulation of E-cadherin and EMT on apoptosis signaling in glioma cells will be a subject of interest and focus in future studies.

β-Catenin, JNK, and ERK have been reported to play a role in the carcinogenic process in several neoplasms 56-58. However, these kinases also participate in cell death induction 24, 25, 29. Our study showed that Cas III-La induced β-catenin phosphorylation at Ser^45^, inhibiting its phosphorylation at Ser^33/37^, and it induced β-catenin accumulation in the nucleus, possibly by blocking proteasomal degradation. The activity of GSK3β is tightly regulated, and it is required to control β-catenin expression. The activity of GSK3β can be regulated through phosphorylation at Ser^9^ by other kinases, like p70 S6 kinase, PKA, RAC, p90^RSK^, and PKC 59. In addition, a suppression of GSK3β activity, either directly by AKT or by Dvl, may mediate the Wnt pathway 60. LiCl induced the accumulation of β-catenin by inducing the inhibitory Ser^9^ phosphorylation of GSK3β 61. LiCl has also been reported to suppress Ser^33^/Ser^37^/Ser^41^, but not Ser^45^ phosphorylation 62. These data suggest that Cas III-La inhibits the activity of GSK3β through its phosphorylation at Ser^9^, which leads to a nuclear accumulation of β-catenin by inhibiting the phosphorylation at Ser^33/37^. Importantly, the inhibition of GSK3β seems to reduce the survival and proliferation of tumor cells and to increase apoptosis by raising the levels of p53-dependent Bax and the cytoplasmatic release of cyt c, with the ensuing activation of caspase-9 and caspase-3 10. GSK3β leads to the inactivation of mdm2, which inactivates p53 63. It has also been demonstrated that GSK3β inhibition suppresses glioma cell migration and invasion by reducing cell polarity 64. Furthermore, GSK3β leads to the phosphorylation of c-jun, blocking its binding to DNA and therefore the synthesis and activity of the transcriptional factor AP-1 65.

Overexpression or accumulation of β-catenin has been proved to promote apoptosis in several cancer lines 24, 25, 29. The antineoplastic effects of the overexpression of β-catenin in rhabdomyosarcoma and osteosarcoma has been linked to a decrease in cell proliferation and apoptotic activity by inhibiting GSK3β 66. Raab et al. showed that Enzastaurin, a PKC inhibitor, induces an accumulation of β-catenin in multiple myeloma cells by blocking the phosphorylation of β-catenin at Ser^33/37^ without affecting phosphorylation at Ser^45^, which prevents its proteasomal degradation. β-Catenin accumulation induces cell cycle arrest by upregulating p21^waf1^, an effect mediated by the endoplasmic reticulum-related unfolded protein response (UPR), but it also promotes apoptosis via c-jun overexpression by p73 induction. Furthermore, β-catenin is accumulated in the nucleus and co-localized with TCF-4. Those authors also found that the amount of β-catenin attached to the membrane is unaltered 67. Inoue et al. reported that the β-catenin/TCF4 complex and JNK induce the transcription of the stress response gene ATF3 and exhibits an antineoplastic effect by repressing migration and invasion in human colon cancer cells 23, 68. ATF3 overexpression reduced migration and the induction of tissue inhibitors of matrix metalloproteinases in glioblastoma 69. However, Kim et al. demonstrated that the overexpression of β-catenin in tumor cells induces apoptosis without transactivating β-catenin/LEF-1, and independently of p53, RB, and Cyc D; however, apoptosis is retarded in cells in which Bcl-xL is overexpressed. These authors suggest that accumulated β-catenin binds other pro-apoptotic proteins through death domain(s) located in Armadillo repeats 70.

On the other hand, it has been reported that Wnt and ERK induced the activation of JNK 71. Wnt activates Rac, which induces the phosphorylation of β-catenin at Ser^191^ and Ser^605^ by JNK, a necessary event for the translocation and nuclear accumulation of β-catenin 72. Additionally, it has been reported that JNK is activated by Dvl 71. JNK activation leads to the phosphorylation of c-jun, which mediates the interaction of Dvl with the β-catenin/LEFs complex 71; this facilitates the formation and stability of the complex c-jun/Dvl/β-catenin/LEFs on the promoter of Wnt target genes. Our study demonstrated that Cas III-La at a dose of 1.5 or 2.5 μg/ml induces the activation of ERK and JNK. This activation of ERK and JNK is consistent with the inhibition of GSK3β and the nuclear accumulation of β-catenin. These results suggest that the Wnt pathway could inactivate GSK3β but also activate JNK, as shown by the nuclear accumulation of β-catenin, further supporting the antineoplastic effect of Cas III-La on the glioma U373 MG cell line. Li et al. proposed that the inhibition or upregulation of the Wnt/β-catenin pathway favors apoptotic death 22; high levels of β-catenin have also been related to an anti-invasive effect 23. Furthermore, it has been demonstrated that AZD2858 (a GSK3β inhibitor) induces the nuclear accumulation of β-catenin, reducing the proliferation and invasion in glioma cells through of upregulation of p53, which regulates the transcription of p21, the Fas ligand, and caspase-3 31, as well as the downregulation of proteins involved in cell adhesion and cell migration, like collagen type I, Laminin, CD44, and fibronectin 31.

**Fig 10:**
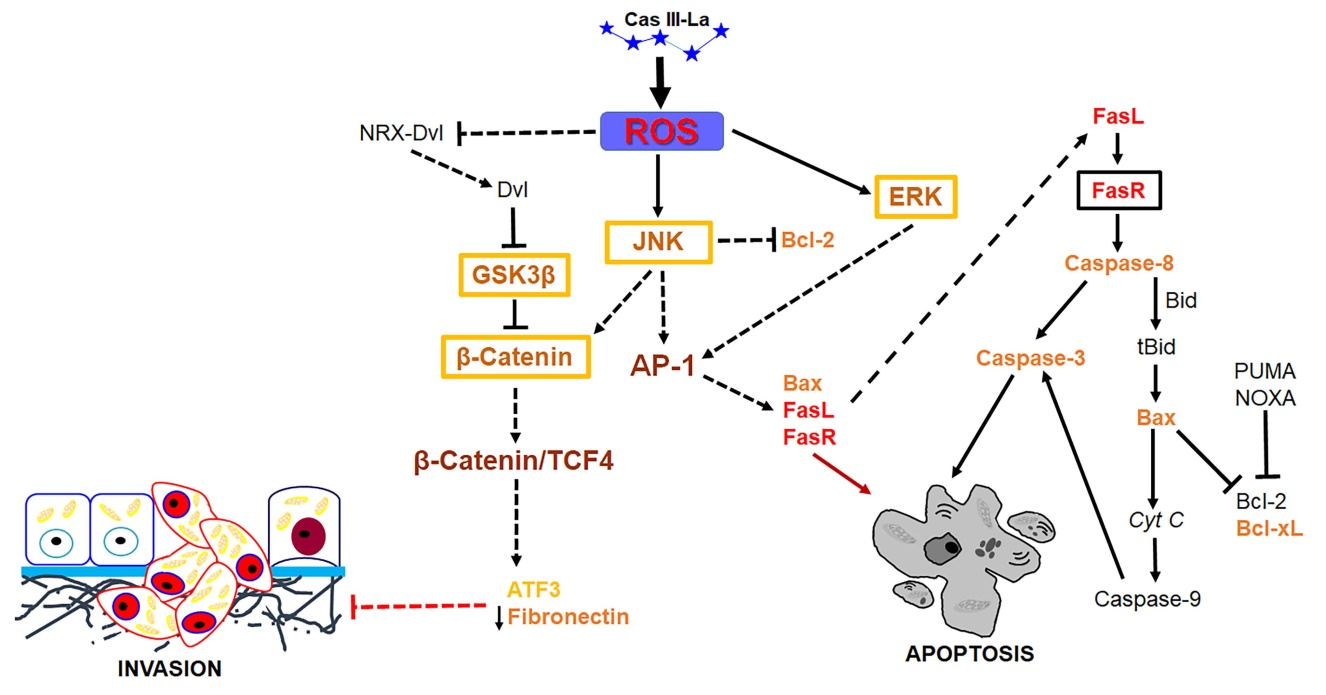
Suggested pathway initiated by Cas III-La to promote the inhibition of the invasive process and the induction of cell death on U373MG glioma cells. Cas III-La can generate reactive oxygen species (ROS), which can release at Dvl from the NRX-Dvl complex. Dvl can inactivate GSK3β, inducing the activation and nuclear accumulation of β-catenin. β-catenin can bind and activate TCF4, which may activate the transcription of ATF3. ATF3 can increase the expression of tissue inhibitors of matrix metalloproteinases, which inhibit MMP2 and MMP9. In addition, ROS can activate ERK and JNK, which activate the AP-1 transcription factor formed by c-jun/c-Fos. AP-1 can induce apoptosis via transactivation of the Bax, FasL, and FasR genes. FasR induces the activation of caspase 8, which activates caspase-3, inducing apoptosis. Also, caspase 8 can cleave the pro-apoptotic protein Bid into tBid, inducing Bax oligomerization and the consequent activation of the apoptosis mitochondrial pathway by releasing cyt c and activating caspase-9, which activates caspase-3. Furthermore, JNK can phosphorylate β-catenin, inducing its nuclear translocation.

Previous studies have shown that ROS may serve as signaling molecules that directly or indirectly activate apoptosis by regulating signaling proteins like β-catenin, JNK, and ERK 22, 23. Our study demonstrated that Cas III-La induced the formation of ROS and activated ERK and JNK, as well as a nuclear accumulation of β-catenin. Furthermore, the inhibition of Cas III-La-mediated ROS generation by NAC not only offset the decrease in cell viability and anti-invasive effect on U373 glioma cells, but it also mitigated the activation of ERK and JNK and the nuclear accumulation of β-catenin, preventing cell death. A possible mechanism by which ROS promote the accumulation and nuclear translocation of β-catenin was proposed by Funato et al. 73. Dvl inhibits the activity of GSK3β and promotes the stability of β-catenin 74. This capacity of Dvl depends on its binding to nucleoredoxin (NRX, a ubiquitous antioxidant), a protein of the thioredoxin family 74. These authors demonstrated that the interaction in the Dvl-NRX complex was blocked in the presence of H_2_O_2_, resulting in the liberation of Dvl and subsequent activation of Wnt/β-catenin by increasing the levels of the β-catenin/TCF nuclear complex 73. Dvl also activates β-catenin accumulation independently of JNK, directed by Wnt/Fzd/Dvl 71. These results suggest that Cas III-La inhibits cell proliferation, migration, and invasion in the U373 cell line, promoting apoptosis, by generating ROS, which could induce the release of Dvl with the subsequent activation and the nuclear accumulation of β-catenin, as well as the activation of ERK and JNK, which activate the AP-1 transcription factor formed by c-Jun/c-Fos. AP-1 can induce apoptosis via transactivation of the Bax, FasL, and FasR genes. Furthermore, JNK can phosphorylate β-catenin, inducing its nuclear translocation (Fig. 10). On the other hand, β-catenin can bind and activate TCF4, which may activate the transcription of ATF3 23, 68 and p53. ATF3 can increase the expression of tissue inhibitors of matrix metalloproteinases, which inhibit MMP2 and MMP9 69. p53 can activate the transcription of pro-apoptotic proteins such as Bax, Puma, Noxa, and the Fas ligand; the later activates apoptosis via FasR 75. In addition to its transcriptional regulatory function, p53 acts as an anti-apoptotic protein in mitochondria. p53 binds and inhibits Bcl-2 and Bcl-xL 76, 77, which induce mitochondrial outer membrane permeabilization and a release of cyt c from mitochondria in cancer cells 78. The U373 cell line has been described to have a point mutation in codon 273 (CGT→CAT, Arg→His) on the exon 5, a mutation 'hotspot' on the p53 gene, which inhibits DNA binding 79. However, this mutant has the capacity to bind and transactivate p53 DNA binding sequences 80, as well as retaining 98% of the conformational structure of the wild-type p53 protein 81. Due to the presence of mutated p53 in the U373 cell line, Cas III-La could exert its antineoplastic effect through a p53-independent mechanism; however, the relation between wild-type p53 and mutated p53 with Cas III-La-induced apoptosis in glioma cell populations should be further studied in the future.

On the other hand, Cas II-gly has been reported to inhibit the Wnt signaling pathway by downregulating the expression of the frizzled class receptor 2 (FZD2) and the metastasis-associated lung adenocarcinoma transcript 1 (MALAT1) while upregulating the expression of miR-17-5p, resulting in apoptosis promotion in CaSki and HeLa cells 82. These differences in the modulation of the Wnt pathway may be due to the type and concentration of copper compounds, as well as the experimental model used in each study.

Previously, we reported that other copper compounds like Cas III-ia inhibits cell proliferation and induces apoptosis and autophagy in C6 rat glioma cells through the formation of ROS, followed by activation of JNK, phosphorylation of c-jun, and expression of Beclin 1, Atg 7, and Bax 16. The pharmacological inactivation of JNK reduced Cas III-ia-induced apoptosis 16.

The administration of Cas III-La in nude mice xenotransplanted with U373 MG cells showed an antitumoral effect, decreasing tumoral volume, as well as the mitotic and cell proliferation indices, with evidences of apoptosis as its mechanism in vivo. No significative weight loss or death were observed neither in control nor treated mice.

The molecular mechanisms through which Cas III-La promoted ROS generation in our study in vitro have not been established. However, other Casiopeinas have been proposed to induce ROS generation. Kachadourian et al. proposed that glutathione (GSH) plays a key role in ROS production by reacting with Cas II-gly. GSH reduces the copper complex in Casiopeinas from Cu^2+^ to Cu, forming the glutathyl radical (GS^•^). GS^•^ deactivation can occur through two pathways: a) by reacting with a second GS^•^, yielding oxidized glutathione (GSSG), or b) by reacting with GSH and oxygen, yielding the superoxide anion (O_2_^-•^) plus GSSG. Superoxide dismutase (SOD) transforms O_2_^-•^ into hydrogen peroxide (H_2_O_2_); H_2_O_2_ reacts with the reduced Casiopeina, yielding the hydroxyl radical (HO^•^). This causes damage to mitochondrial DNA, deregulating the translation of proteins of the mitochondrial respiratory chain and increasing oxidative stress, which leads to mitochondrial dysfunction by decreasing the levels of GSH (a key antioxidant molecule) and increasing those of H_2_O_2_ 14. Garcia-Ramos et al. had proved that Cas II-gly, Cas III-ia, and Cas III-Ea induce apoptosis through the mitochondrial pathway in the neuroblastoma SK-N-SH cell line, generating O^-•^_2_ and H_2_O_2_ by oxidizing GSH 8. Our study demonstrated the formation of ROS and their localization within mitochondria. These results suggest the mitochondria-mediated production of ROS, which are associated with mitochondrial membrane potential collapse and the induction of the mitochondrial apoptotic pathway 83, 84. We previously reported that other copper compounds such as Cas II-gly induce apoptotic cell death, both by caspase-dependent and -independent pathways, by generating ROS 12. Furthermore, herein we demonstrated that Cas III-La inhibits the enzymatic activity of SOD1, SOD2, and catalase 16.

On the other hand, the lower cytotoxic effect of some Casiopeinas (Cas III-ia, Cas II-gly, and Cas III-Ea) on cultures of human peripheral blood lymphocytes and fibroblasts showed the selectivity of these compounds for tumor cells over non-transformed cells 8, 10. To confirm the specific cytotoxic effect of Cas III-La on other glioma cells besides the U373 MG line, the effect of the drug at a dose of 0, 0.5, 0.75, 1.5, or 2.5 μg/ml on fibroblasts and on LN18, U87, and T96G human glioma cells, as well as on C6 rat glioma cells, was determined by the MTT assay after 24 h of incubation. Cell viability was 100% for fibroblasts treated with 0.5, 0.75, or 1.5 μg/ml of Cas III-La, and it was 90% for cells treated with 2.5 μg/ml. T96G and C6 glioma cells were highly sensitive to the cytotoxic effect of the drug since the lowest dose, and both showed a dose-dependent effect in the whole dose-range. LN18 and U87 cells showed a dose-dependent effect since a dose of 0.75 and 1.5 μg/ml, respectively (Supplementary Table 1). Furthermore, it has been demonstrated that Cas III-La has a lower effect on the viability of human lymphocytes and macrophages 85. These results suggest that Cas III-La has an antineoplastic effect on several glioma cells, and it is specific against malignant cells.

## Conclusions

Our study demonstrates that Cas III-La induces the production of reactive oxygen species, promoting the inhibition of GSK3β, the activation of JNK and ERK, as well as the accumulation of β-catenin, which in turn induce apoptosis. Our results indicate that Cas III-La is a promising chemotherapeutic agent against glial malignant tumors.

## Supplementary Material

Supplementary figures and table.

## Figures and Tables

**Table 1:** Effect of Cas III-La on the cell cycle of glioma U373 cells.

Cas III-La (μg/ml)	Sub-G_0_ (%)	G_0_ / G_1_ (%)	S/M (%)
0	2.48 ± 0.59	73.94 ± 1.40	22.79 ± 2.32
1.5	43.29 ± 5.38^***^	34.9 ± 4.69^***^	13.3 ± 3.94^***^
2.5	73.36 ± 0.83^***^	11.05 ± 0.53^***^	12.22 ± 5.92^***^

^1^ Results are expressed as mean ± SD. Statistical significance was calculated by comparing untreated cells with Cas III-La-treated cells (1.5, 2.5 μg/ml). ***P ≤ 0.0001.

## Abbreviations

GBM: Glioblastoma multiforme
Cas III-La: Casiopeina III-La
cyt c: cytochrome c
ROS: Reactive oxygen species
NAC: N-acetyl-L-cysteine
CNS: Central nervous system
WHO: World Health Organization
PDGR: Platelet-derived growth factor receptor
EGFR: Epidermal growth factor receptor
VEGFR: Vascular endothelial growth factor receptor
MAPK: Mitogen-activated protein kinase
mTOR: Mammalian target of rapamycin
PI3K: Phosphoinositide 3-kinase
GSK3β: Glycogen Synthase Kinase 3 Beta
MMP-9: Matrix metalloproteinase-9
ERK: Extracellular-signal-regulated kinase
NF-κB: Nuclear factor kappa-light-chain-enhancer of activated B cells
AP-1: Activator protein 1
HIF-1α: Hypoxia-inducible factor 1-alpha
ATF4: Activating transcription factor 4
Dvl: Dishevelled
rTRAIL: Recombinant TNF receptor death-inducing ligand
Bcl-2: B-cell lymphoma 2
Bcl-Xl: B-cell lymphoma-extra large
PCNA: Proliferating cell nuclear antigen
MMP2: Matrix metalloproteinase-2
MMP9: Matrix metalloproteinase-9
DMEM: Dulbecco's modified Eagle's medium
FBS: Fetal bovine serum
MTT: 3-[4,5-dimethylthiazol-2-yl]-2,5-diphenyltetrazolium bromide
PBS: Phosphate buffer solution
PFA: Paraformaldehyde
DCFH-DA: 2′,7′-dichlorofluorescein diacetate
