# Supplementary material for: Anti-proliferative, pro-apoptotic and anti-invasive effect of the copper coordination compound Cas III-La through the induction of reactive oxygen species and regulation of Wnt/β-catenin pathway in glioma
Source: J Cancer. 2021 Jul 25;12(19):5693–711. doi: 10.7150/jca.59769 (PMC8408120; doi:10.7150/jca.59769)
Supplement: Supplementary file 1 — Supplementary figures and table. [file jcav12p5693s1.pdf]

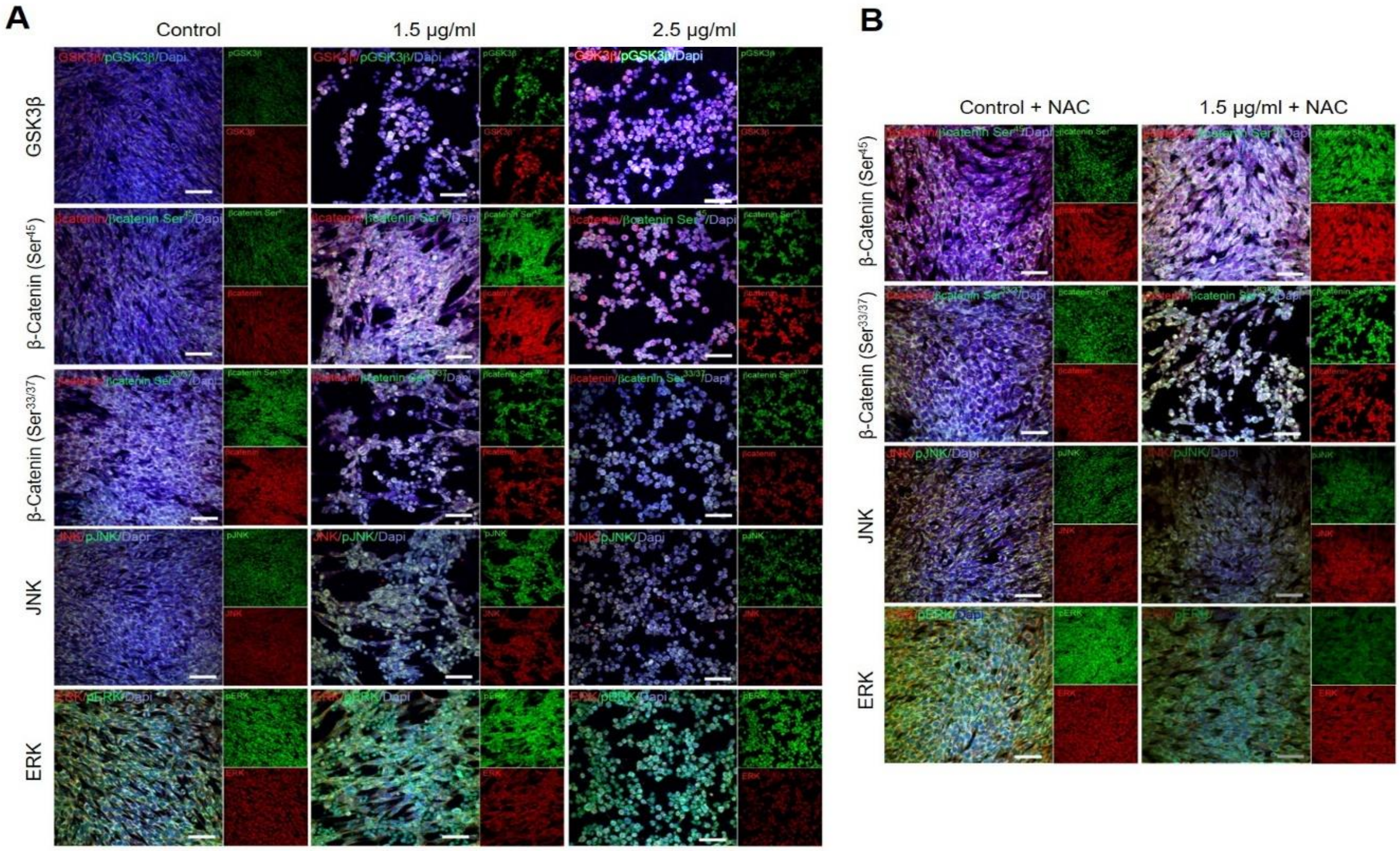

Supplementary Figure 1A

Cas III-La leads to the activation of JNK and ERK, and the nuclear translocation of β-Catenin, related to the induction of ROS. (a) Treatment with Cas III-La induce the inactivation of GKS-3β as we observed with the increase of pGKS-3β (fist row, green), with a consecutive increase of pβ-Catenin (Ser^{45}) (second row, green) but not for pβ-Catenin (Ser$^{33/37}$). In addition, the phosphorylated forms of JNK and ERK are increased (fourth and fifth rows, in green). (b) NAC inhibits the accumulation of β-Catenin and the activation of JNK and ERK. pβ-Catenin (Ser^{45}), pβ-Catenin (Ser$^{33/37}$), pJNK (Thr^{183} and Tyr^{185}) and pERK (Tyr^{204}) are in green. Total GKS-3β, β-Catenin, JNK and ERK are in red and all nucleus are counterstained with Dapi (blue). Bar = 50 μm.

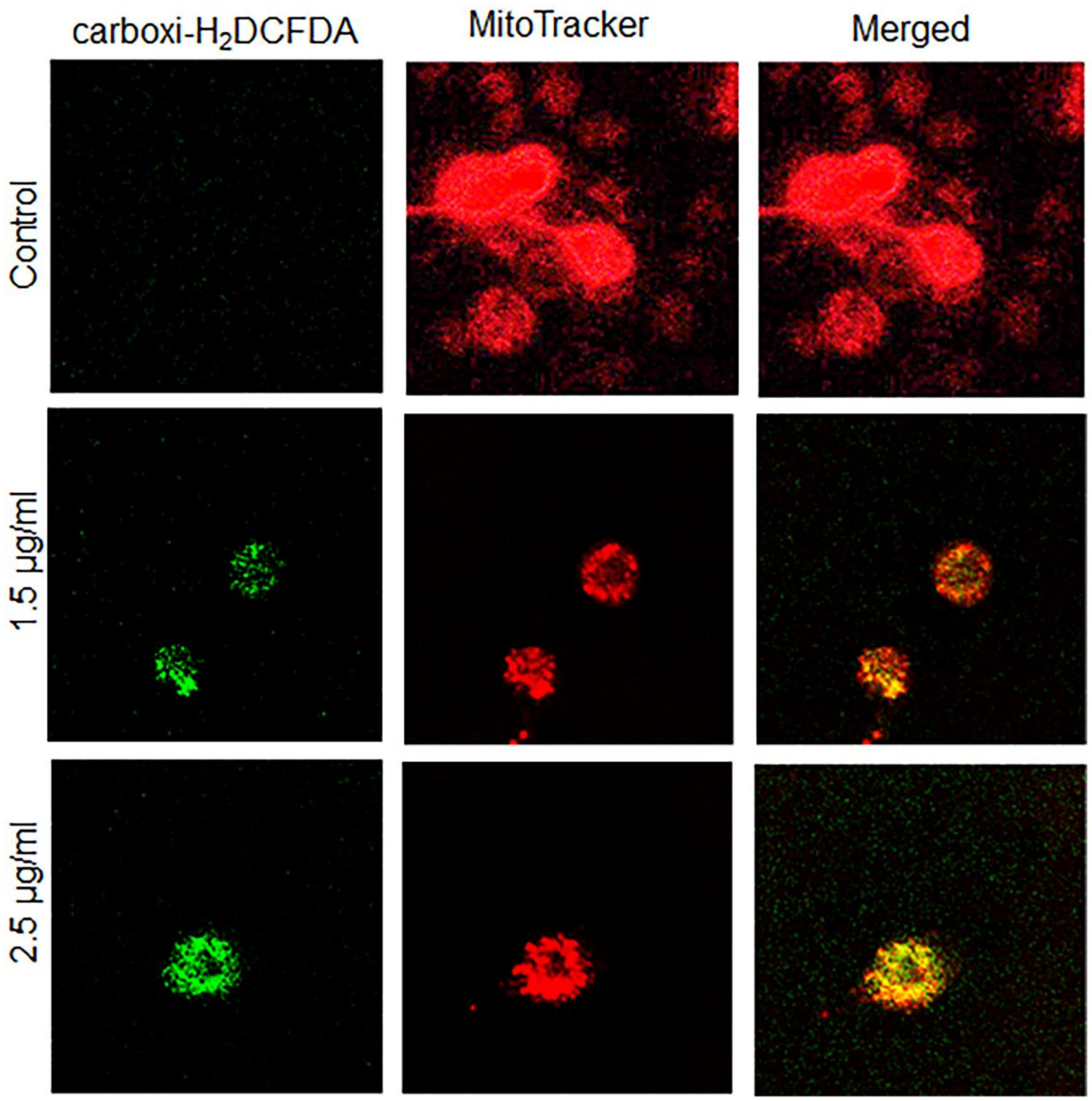

Supplementary Figure 2

Cas III-La induces the generation of ROS in mitochondria. The presence of ROS in mitochondria was also evinced by the co-localization of green fluorescence (carboxi-H_2DCFDA)/red fluorescence (MitoTracker red) in Cas III-La treated cells.

Supplementary Table 1. Effect of Cas III-La on cell viability percentage on normal and glioma cells

Cells	Cas III-La (μg/ml)				
	**0**	**0.5**	**0.75**	**1.5**	**2.5**
Fibroblast	100 ± 4.6	98.10 ± 2.3	97.59 ± 3.4	93.97 ± 3.5	90.40 ± 1.4***
T96G	100 ± 3.3	63-01 ± 2.9 ***	55.02 ± 1.1***	46.86 ± 1.9***	19.12 ± 1.3***
LN18	100 ± 2.9	99.40 ± 3.8	91.47 ± 3.4***	37.50 ± 1.5***	29.30 ± 2.7***
U87	100 ± 3.8	100 ± 3.6	100 ± 4.5	75.72 ± 1.6***	67.00 ± 3.4***
C6	100 ± 1.7	90.45 ± 1.4***	80.90 ± 2***	43.20 ± 2.9***	22.04 ± 1.2***

Results are expressed as mean ± SD. Statistical significance was obtained by comparing untreated cells versus Cas III-La treatment 0.5, 0.75, 1.5 and 2.5 μg/ml respectively. ***p≤0.0001
